# Understanding time–activity curve and time‐integrated activity variations in radiopharmaceutical therapy challenge: Experience and results

**DOI:** 10.1002/mp.70043

**Published:** 2025-09-26

**Authors:** Oleksandra V. Ivashchenko, Jim O'Doherty, Deni Hardiansyah, Elisa Grassi, Johannes Tran‐Gia, Johannes W. T. Heemskerk, Eero Hippeläinen, Mattias Sandström, Marta Cremonesi, Gerhard Glatting

**Affiliations:** ^1^ Department of Nuclear Medicine and Molecular Imaging University Medical Center Groningen Groningen The Netherlands; ^2^ R&D Collaborations Siemens Medical Solutions Malvern Pennsylvania USA; ^3^ Department of Radiology and Radiological Science Medical University of South Carolina Charleston South Carolina USA; ^4^ Department of Radiography & Diagnostic Imaging University College Dublin Dublin Ireland; ^5^ Department of Physics, FMIPA Universitas Indonesia Depok Indonesia; ^6^ Medical Physics Unit Azienda USL‐IRCCS di Reggio Emilia Reggio Emilia Italy; ^7^ Department of Nuclear Medicine University Hospital Würzburg Würzburg Germany; ^8^ Department of Radiology Leiden University Medical Center, Leiden South Holland The Netherlands; ^9^ Department of Physics University of Helsinki and Helsinki University Hospital Helsinki Finland; ^10^ Department of Radiology Uppsala University Uppsala Sweden; ^11^ Radiation Research Unit European Institute of Oncology Milan Italy; ^12^ Department of Nuclear Medicine Ulm University Ulm Germany

**Keywords:** dosimetry, radiopharmaceutical therapy, time–activity curve

## Abstract

**Background:**

The process of determining/calculating the time–activity curve (TAC) for radiopharmaceutical therapy (RPT) is generally heavily dependent on user‐ and site‐dependent steps (e.g., the number and schedule of measurement points to be used, selection of the fit function), each having a notable effect on the determination of the time‐integrated activity coefficient (TIAC) and thus on the calculated absorbed dose. Despite the high clinical importance of absorbed doses, there is no consensus on the methodology for processing time–activity data or even a clear understanding of the influence of various uncertainties and user‐dependent variations in personalized RPT dosimetry on the accuracy of TAC calculations.

**Purpose:**

To address this critical unmet need, the **t**ime–**a**ctivity **c**urve and **t**ime‐**i**ntegrated activity variations (TACTIC) AAPM Grand Challenge was designed to explore the variations in TAC modeling for RPT applications.

**Methods:**

Launched in January 2023, the TACTIC challenge consisted of three phases: i) warm‐up phase (phase 0, to gain familiarity with the logistics and the modalities of the challenge), ii) TAC fitting based on data from individual patients (phase 1, rated to determine winner 1), and iii) TAC fitting using population‐based data (phase 2, rated to determine winner 2). Based on the distributed synthetic biokinetic data of [^177^Lu]Lu‐PSMA‐617 RPT (kidney, blood, and tumor), participants were asked to model the TAC and calculate the TIAC values for each of these tissues to the best of their ability. In addition, participants were requested to submit information about the fit function and fit optimization parameters. The best‐performing team in each phase was determined on the basis of total root‐mean‐square error (RMSE) value across all three tissues.

**Results:**

A total of 132 teams from over 30 countries registered for this data‐driven challenge, of which 95 individual groups submitted their results throughout the challenge. By presenting participants with an identical set of measurement points previously generated from measured biokinetic data and providing additional a priori information about the procedure at different stages of the challenge, we could assess the degree of variation within the TIAC estimation. We investigated which of the commonly used TIAC estimation methods performs best and could therefore be used to harmonize TAC modeling in RPT dosimetry.

**Conclusion:**

The results of the TACTIC challenge demonstrate the large variability in TAC fitting despite the participants receiving identical input data. This highlights the fundamental role of TAC fitting methodology selection in the calculation of absorbed doses in RPT and successfully raises awareness of the need for greater harmonization in dosimetric approaches.

## 1 INTRODUCTION

The process of radiopharmaceutical therapy (RPT) dosimetry is inherently complex, involving several critical components. It requires precise quantitative measurements of biokinetics data, such as segmented quantitative images from modalities such as SPECT/CT. Additionally, it requires a thorough understanding of the physical processes underlying radiation deposition, including the radionuclide's decay scheme and the spatial relationship between source and target organs. Accurate and reliable software is also essential for delineating target regions or volumes of interest and for the precise estimation of time–activity curves (TAC) and time‐integrated activity coefficient (TIAC—represents the cumulated activity in a source region over time, normalized to the injected activity, therefore is expressed in units of time). The SNMMI ^177^Lu Dosimetry Challenge 2021^1^ was one of the first coordinated efforts to draw international attention to the fact that RPT dosimetry is overwhelmingly user and software‐dependent.^2^ Because of the high complexity of the RPT dosimetry workflow and the limited number of biokinetic data sets (i.e., two patients without a ground truth) included in the SNMMI challenge, the results mainly emphasize the fact that there is a wide variation in RPT dosimetry methods, and that TAC fitting and integration have a considerable effect on dosimetry calculation, yet did not provide a clear assessment of the contributions of individual steps within the RPT dosimetry workflow.

Consequently, the **t**ime‐**a**ctivity **c**urve and **t**ime‐**i**ntegrated a**c**tivity variations (TACTIC) AAPM Grand Challenge 2023 was designed to focus on variations in TAC as a crucial element in RPT dosimetry.^3^ We aimed to answer some fundamental questions about TAC fitting, such as, if different users receive the same set of measurement points and identical a priori data about the treatment, will the calculated TIAC differ? How different will their results be? What does the variation depend on? These questions are critical for developing, optimizing, and eventually harmonizing personalized RPT dosimetry methodology but have not yet been sufficiently answered.

Previous work has already demonstrated that the function chosen for interpolation and extrapolation of the time–activity data, together with the number of data samples and their measurement uncertainty, strongly influence the calculated TIAC^4^, ^5^, ^6^ in turn directly affecting the absorbed dose calculation. These high levels of user dependency in RPT dosimetry hamper the development of dosimetry‐guided treatment planning. Ultimately, they leave the major advantage of RPT over chemotherapy or biologic therapy unexploited^7^: namely, the possibility to immediately monitor systemic applications of radiopharmaceuticals by imaging and thus optimizing such treatments for well‐defined patient populations or even individual patients.

Keeping the aforementioned variations in mind, the TACTIC challenge was designed to evaluate the degree of variations within the TAC acquisition/ TIAC estimation within the RPT community and to investigate which of the commonly used TIAC estimation methods performs best in a controlled test environment. Providing participants with synthetically generated biokinetic data for [^177^Lu]Lu‐PSMA‐617 and a priori information about the data, we evaluated the influence of the preferred time distribution for measurement points (e.g., with or without late measurement time points beyond 150 h post‐injection) as well as a possible added value of the use of population‐based information (intra‐ and inter‐individual variability) on the accuracy of calculated TIAC values for a series of chosen tissues (i.e., kidney, blood, and tumor lesions). This report provides a detailed summary of the structure and proceedings of the TACTIC AAPM Grand Challenge 2023 and an analysis of the collected data.

## 2 Challenge Design

### 2.1 Data

The challenge utilized 25 synthetic biokinetic datasets (i.e., ground truth time–activity data) of renal, tumor, and blood activity measurements derived from high‐quality clinical data from a 63‐patient cohort who had received [^177^Lu]Lu‐PSMA‐617 therapy, with permission from the authors of reference 8.^8^ For each patient, at least five post‐therapeutic SPECT/CT scans (two field of view) had been acquired at approximately 2 ± 1, 19 ± 1, 43 ± 1, 66 ± 1, and 160 ± 24 h after injection of [^177^Lu]Lu‐PSMA‐617. For each scan, kidney, and tumor uptake were evaluated, and blood samples were collected to determine activity concentration near the SPECT/CT time points.

The synthetic data were generated through a multi‐step process:

1. **Biokinetic data fitting**: The parameters of various sum‐of‐exponential functions (SOEFs) with different parameterizations were fitted to the biokinetic datasets of [^177^Lu]Lu‐PSMA‐617 activity in the kidney, tumor, and blood separately using the non‐linear mixed‐effects (NLME) modeling framework, assuming a proportional error model for measurement uncertainties (intra‐individual variability).^9^ The optimal SOEFs were identified for each biokinetic dataset through population‐based model selection based on the goodness‐of‐fit test and Akaike weight.^6^ The output of this data fitting was the optimal SOEF, the estimated population parameters of fixed effects and random effects (inter‐individual variability), the estimated proportional intra‐individual variability noise, and the TACs for all patients and organs. The NLME population fitting approach was chosen in this study as it has been shown to perform better than the individual fitting approach in estimating TIAC.^9^
2. **Selection of patients**: 25 patients were randomly selected from the population of 63 patients for the challenge. The TACs and TIACs of these patients (generated from the optimal SOEF and the estimated population parameters in Step 1) were considered the ground truth for evaluation.
3. **Biokinetic uncertainty (intra‐individual variability) simulation**: A random Gaussian‐distributed proportional error was added to the ground truth TACs according to the magnitude determined in step 1 (NLME modeling, i.e., FSD = 7.9% for kidneys, 18% for tumor, and 7.2% for blood). This step aimed to make the synthetic data reflect actual patient variability while accounting for measurement uncertainties.

The type of data and sampling time points are detailed in Appendix 1. In the following, the synthetic data sets obtained based on the biokinetic model will often be referred to as “patients” for simplicity. Using synthetic patient data allowed us to compare to a ground truth, and provided freedom in selecting imaging time points.

### 2.2 Structure

**FIGURE 1:**
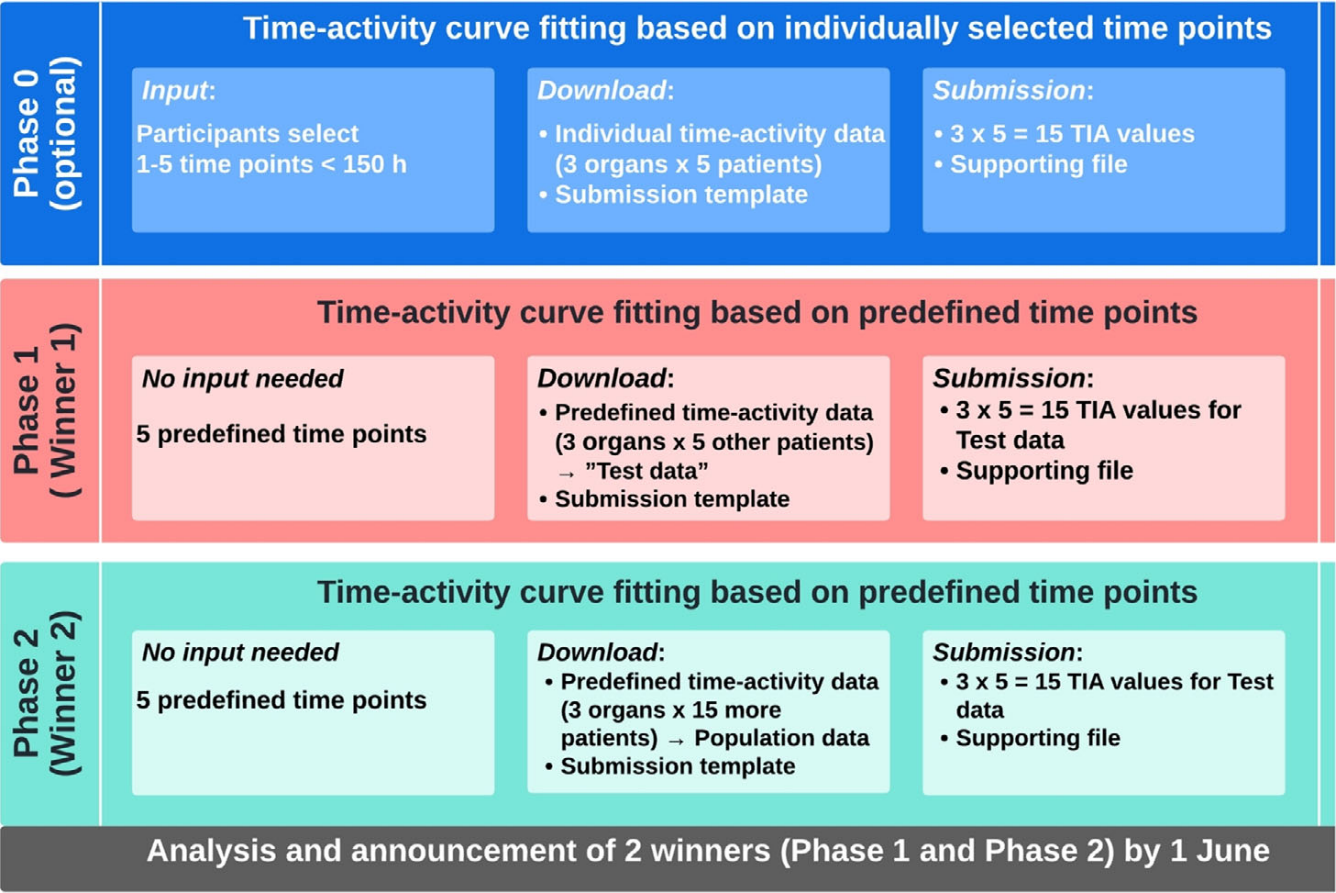
Schematic illustration of the grand challenge.

The challenge was structured into two complexity levels: an unsupervised “warm‐up” phase (Phase 0) and weakly supervised competition phases (Phases 1 and 2) for TAC and TIAC estimations, as outlined in Figure 1. With a dataset comprising 25 synthetic patients, ground truth data was available for all cases. This ground truth includes the SOEF and its corresponding parameters for each organ, providing a reliable basis for evaluating the accuracy of the estimations.

The dataset size was intentionally limited to highlight the educational objective of demonstrating the benefits of incorporating population‐based prior knowledge, rather than optimizing model fitting with a larger cohort. In phases 0 and 1, only five patients were included for comparison, while in phase 2 the dataset was expanded by 15 additional patients (20 in total), providing sufficient statistical power for meaningful comparisons while maintaining the training focus.

#### 2.2.1 Warm‐up phase (“phase 0″, unrated)

Each participant was informed that they will receive biokinetic data of [^177^Lu]Lu‐PSMA‐617 in tumor, kidney, and blood for five patients (patients 1 to 5). Participants were asked to provide the desired measurement schedule (up to five time points in the range of 1 to 150 h after injection). Next, they were provided with the corresponding synthetic time–activity data and asked to calculate and submit the TIAC for each organ for each patient and to provide fit functions and parameters to justify their specific approach via the questionnaire. Subsequently, the scoring parameters of the submitted data were evaluated (see scoring parameters below) and used to determine the leaderboard of the 0th phase. This phase was designed to introduce the general methodology of the challenge to the participants, and to provide a general sense of the influence of the sampling scheme and fit functions on the outcome

The complete list of questions can be found in Appendix 2. The same questionnaire was used for all phases.

#### 2.2.2 First competition phase (“Phase 1″; rated to determine winner 1)

In Phase 1 of the challenge, the pharmacokinetic measurement scheme was standardized to a fixed set of five data points for all participants, corresponding to the original SPECT/CT measurement time points from the clinical data used for modeling (see Section 2.1 Data). Additionally, five new synthetic patient datasets (patients 6 to 10) were provided to participants. Participants were tasked with completing a questionnaire and submitting their estimated TIACs, which were then evaluated (criteria described in Section 2.3) to determine the winner of Phase 1.

#### 2.2.3 Second competition phase (“Phase 2″; rated to determine winner 2)

An additional dataset of 15 synthetic patients (i.e., patients 11 to 25), with biokinetic data at the same time points as that in phase 1, was shared with the participants. This dataset was provided as an additional source of information for the TAC fitting for synthetic patients 6 to 10, i.e., to include population‐based information. The participants were encouraged to recalculate and resubmit TIAC for patients 6 to 10. The results from the TIAC analysis of synthetic patients 11 to 25 were not scored.

All participants received an overview of the literature and methods that can be used to incorporate the population‐based information.^10^, ^11^, ^12^, ^13^ However, no suggestion or recommendation on the exact method was made by the organizers. Additionally, the participants could choose to ignore the recommendation to incorporate the population‐based data and could use another fitting strategy if they had reason to believe that it performed better.

### 2.3 Performance evaluation

In all phases of the challenge, the primary parameter used to evaluate participants' performance was the root‐mean‐square error (RMSE) of the TIAC, generally expressed in units of MBq∙h. The RMSE was summed over all patients for kidneys, tumor lesions, and blood:

(1) $$RMSE=\sqrt{\frac{1}{5}\sum_{i=1}^{5}{\left[TIA_{patient,i}-TIA_{groundtruth,i}\right]}^{2}}\;$$

where i is the patient number. The total RMSE was calculated as an equally weighted sum of the tissue RMSEs. In addition, the information from the questionnaire (Appendix 2) was used to verify the validity of the submitted TIAC values. Therefore, any submissions that omitted information about the fit function, objective function, and explicit fit parameters were disqualified from the challenge.

The participant with the lowest total RMSE value over all tissues and patients was declared the winner of the phase. In the event of a tie (taking into account two significant figures after a decimal point), the participant with the lowest RMSE value for the tumor only would be given priority. For the sake of clarity, all RMSE values below are expressed in h or h/mL (tumor).

### 2.4 Statistical testing

To evaluate quantitative performance differences across the challenge phases, we performed statistical analyses to assess potential reductions in average RMSE both within and between phases. Since the TIAC RMSE data for all tissues were not normally distributed (Shapiro–Wilk test, *p* < 0.001 for phases 0, 1, and 2 across all tissues), we used the non‐parametric Mann–Whitney U test for unpaired data. This test was applied to overall RMSE values, as well as tissue‐specific RMSE values (kidney, tumor, and blood).

For cross‐phase comparison, the null hypothesis assumed no difference in mean RMSE between a given phase and its preceding phase. For tissue‐specific comparisons within a phase, the null hypothesis assumed equal mean RMSE values across tissue types.

For participants who submitted results in multiple phases, we also performed the Wilcoxon signed‐rank test for paired data, using the same null hypotheses as in the Mann–Whitney tests.

## 3 RESULTS

### 3.1 Number of participants and geographical distribution

**FIGURE 2:**
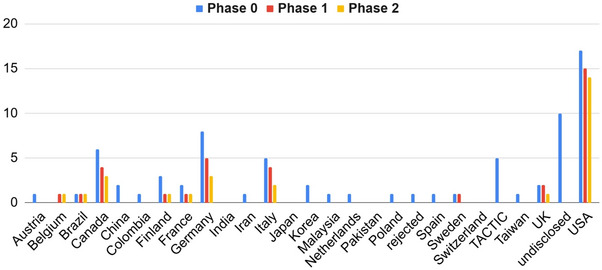
TACTIC challenge country participation by phase.

A total of 132 individual teams from over 30 countries registered for the challenge. Of these, 73 (55%) requested and 35 (26%) submitted the data for phase 0, 33 (25%) and 28 (21%) participated in the phase 1 and phase 2 competition phases, respectively. A total of 21 teams participated in all phases, with 56 teams participating in at least one phase. Additionally, each team that participated in Phase 2 also participated in Phase 1. A breakdown of participation by country can be observed in Figure 2 (See Table 1).

### 3.2 Phase 0

During phase 0, we received 73 requests for data at freely chosen sampling time points. Of these requests, 39 teams submitted TIAC calculations and the corresponding questionnaire (Figure 2). Nine teams were disqualified, four for incomplete questionnaires and five as members of the organization team, who were permitted to take part out of interest, but not as official competitors. As is observed in Figure 2, a total of 10 teams decided not to disclose their team origin.

#### 3.2.1 Requested sampling scheme

**FIGURE 3:**
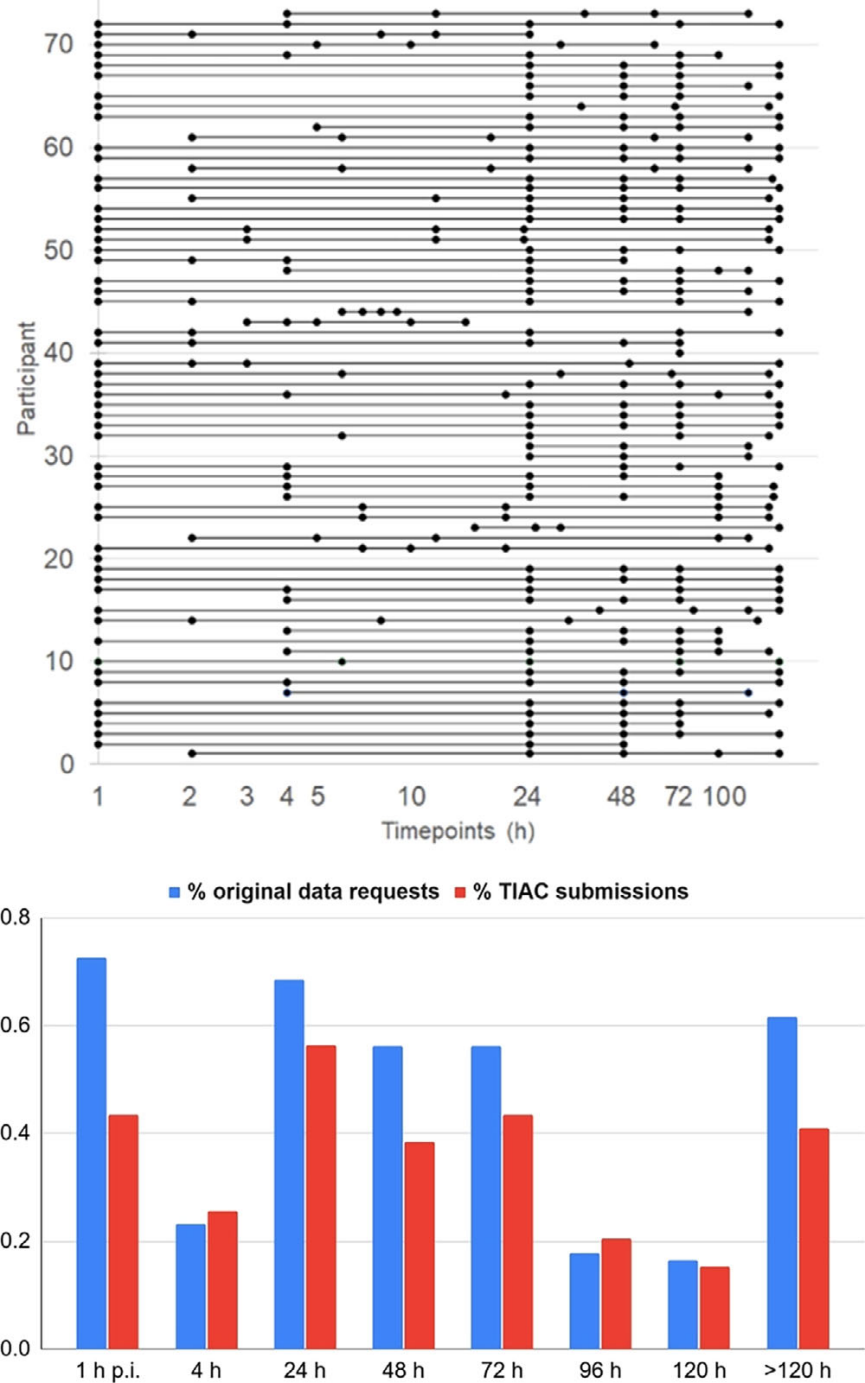
Distribution of the data points requested in phase 0 by all participating teams.

For the user‐defined sampling scheme, 65 teams (89%) requested 5 data points, and 2, 4, and 2 teams (2.7%, 5.4%, and 2.7%) requested 4, 3, and a single data point, respectively. Out of the teams that submitted TIAC calculations, the latest time point requested was on average 127 ± 35 h p.i., while the median time point per requested schedule was 35 ± 19 h p.i. The most frequently requested points were at 24 h (68.5%), 48 h (56.2%), and 72 h (56.2%) and 140 to 150 h (61.6%). A summary of the frequency of requested points and a full overview of the requested timepoints for each team can be observed in Figure 3.

#### 3.2.2 Questionnaire phase 0

Apart from the competitive element of the challenge, the questionnaire responses were of particular scientific interest, e.g. on the fitting method. Danieli et al.^14^ previously demonstrated that TIAC values ​​depend on the curve‐fitting methodology used. Furthermore, multiple authors^5^, ^15^, ^16^ have emphasized the importance of the imaging schedule, uncertainty budget, and model selection criteria using synthetic data for [^177^Lu]Lu‐PSMA. The complete questionnaire can be found in Appendix 2. Although this section presents figures for all phases of the challenge, the results for each phase are discussed independently for clarity.

**FIGURE 4:**
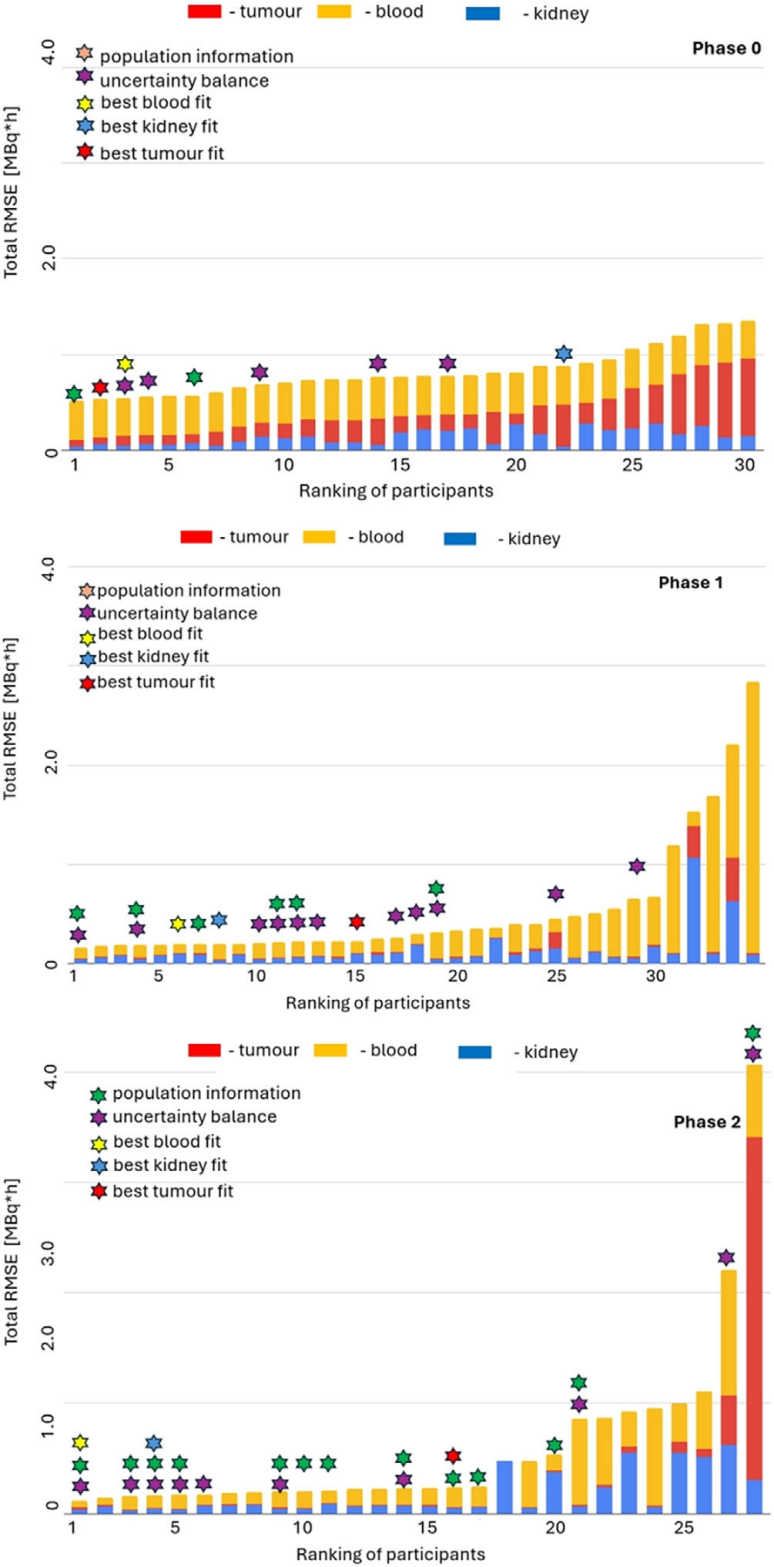
RMSE results for all participants for phase 0, phase 1, and phase 2 of the challenge (unit: h for kidney/blood and h/mL—tumor).

Of the 39 teams who submitted TIAC calculations in phase 0, only 5 (17 %) incorporated a data uncertainty model into the fit (Figure 4); the rest did not (or did not answer positively). In addition, 9 (30 %) performed a visual inspection only, 6 (20 %) used the Akaike information criterion (AIC—a test that statistically compares the quality of a set of models to each other), 3 (10%) employed RMSE, and 9 (30%) *R* ^2^ as one of the checks for the goodness of fit. Only 2 (6.7%) teams took population information into account, potentially from literature or from experience. This population information was used to determine appropriate fitting curves and set initial parameters (i.e., peak tissue uptake and half‐life) or boundary limits for the fitting process. One team used population‐based information about kidney biokinetics to select the appropriate schedule of the time points, yet did not incorporate this data into the fit itself. An overview of used fit functions can be found in Table 2.

**TABLE 1:** Overview of participation rates per phase of the TACTIC challenge.

	All challenge	Phase 0	Phase 1	Phase 2
Registration	132	73	n/a	n/a
Submissions	104(95^*^)	40(35^*^)	36 (33^*^)	28 (27^*^)

* eligible submissions, with all required information provided.

**TABLE 2:** Fit function per organ in phase 0 (warm‐up), competition phases 1 and 2.

**Fit/regression function**	**Kidney**	**Blood**	**Tumor**
Mono‐exponential	2*/3**/4***	6/1/7	6/5/5
bi‐exponential	19/17/9	13/20/8	12/17/10
3‐exponential	2/6/8	3/5/6	6/3/4
4‐exponential	1/0/0	1/1/0	0/0/0
Convolution of mono‐exponential fit with blood	0/1/1	0/0/0	0/1/1
Convolution of gamma variance with blood	0/1/1	0/0/0	0/1/1
Trapezoid	2/1/1	2/2/2	1/1/1
Trapezoid fit combined with mono‐exponential	4/1/1	3/1/2	4/2/2
Trapezoid fit combined with bi‐exponential	0/0/1	0/0/1	0/0/1
Single compartment model	0/1/2	0/1/1	0/1/1
Two‐compartment model	0/1/0	1/1/0	0/2/0
Log fit	1/0/0	1/0/0	1/0/0
Association‐dissociation	0/0/0	0/0/0	1/0/0
3rd order polynomial	0/0/0	1/0/0	0/0/0
Custom formula	0/1/0	0/0/0	0/0/0

* phase 0.

** phase 1.

*** phase 2.

For every team, organ‐specific RMSE values were calculated and summed to determine the total error, which was then used to establish the leaderboard for this phase. The final ranking and basic statistics are available in Table 3 and Figure 4. The median RMSE for the three target tissues across all participants in Phase 0 was 0.77, with a minimum value of 0.52, and a maximum value of 1.36. The top‐performing team, European Radiation Dosimetry Group (EURADOS), used 5 data points (1, 4, 20, 96, and 140 hours p.i.), and achieved a total RMSE of 0.52. In terms of organ‐specific performance, EURADOS tied for first place in the kidney category (RMSE 0.40), ranked second for the tumor (RMSE 0.68 vs. 0.64 for the best score), and came in 13th for RMSE‐blood (RMSE 0.41 vs. 0.39 for the best score). RMSE values for blood were consistent across all teams, with Phase 0 showing the highest median RMSE among all tissues at 0.41 (Table 3).

**TABLE 3:** Average RMSE performance per phase of the challenge (unit: H or h/mL). Since the data is not normally distributed, median, minimum, and maximum value is provided per phase of the challenge.'

Error type (median (min, max))	Phase 0	Phase 1	Phase 2	Phase 2^*^
RMSE total [h]	0.77 (0.52, 1.36)^**^	0.26 (0.16, 1.69)	0.23 (0.12, 4.07)	0.23 (0.12, 1.1)
RMSE kidney [h]	0.13 (0.04, 0.28)	0.08 (0.04, 1.07)	0.07 (0.03, 0.63)	0.07 (0.03, 0.63)
RMSE tumor [h/ml]	0.18 (0.06, 0.81)	0.01 (0.01, 0.32)	0.01 (0.01 3.11)	0.01 (0.01 0.1)
RMSE blood [h]	0.41 (0.4 0.43)	0.15 (0.09 1.57)	0.15 (0.06 1.14)	0.15 (0.06 0.88)

* excluding outlier teams (*N* = 2).

** median (minimum, maximum)

From literature, it is known that the timing of post‐therapy imaging has a significant impact on post‐therapy dosimetry in RPT.^16^, ^17^, ^18^, ^19^ Therefore, we evaluated whether RMSE‐based performance varied depending on the time points used by the teams. Specifically, we compared the results of teams whose median requested time point was before versus after 48 h post‐injection, since 48 h was the most common median time point among participants in Phase 0. Using a Mann‐We found no significant difference in total or tissue‐specific RMSE values between these groups (*p* = 0.147).

Next, we examined the effect of including late time points. Approximately 40% of Phase 0 participants did not request biokinetic data beyond 120 h post‐injection. Comparing these groups, teams that incorporated data beyond 120 h showed significantly better RMSE values for total (*p* = 0.041), kidney (*p* = 0.022), and blood (*p* = 0.047) measurements (Table 4). No significant relationship was observed for tumor tissue, which may indicate that the effective half‐life of tumor uptake stabilizes before this later imaging phase.

**TABLE 4:** Statistical test results for RMSE values (units: H ‐kidney/blood and h—Tumor) across Phases 0 to 2 of the challenge, as detailed in the statistical analysis section of the manuscript.

	**Performance metric evaluated**			
**Performance**	**RMSE total**	**RMSE kidney (h)**	**RMSE blood (h)**	**RMSE tumor (h/mL)**
Phase 0, effect of median time point <48h	Not significant, *p* = 0.147	Not significant, *p* = 0.233	Not significant, *p* = 0.257	Not significant, *p* = 0.322
Phase 0, effect of late time points >120h	Significant improvement, *p* = 0.041	Significant improvement, *p* = 0.022	Significant improvement, *p* = 0.047	No significant improvement, *p* = 0.142
Phase 1, effect of uncertainty budget	No significant improvement, *p* = 0.221	No significant improvement, *p* = 0.092	No significant improvement. *p* = 0.78	No significant improvement. *p* = 0.404
Phase 2, effect of uncertainty budget	Significant improvement, *p* = 0.037	Significant improvement, *p* = 0.026	No significant improvement. *p* = 0.63	No significant improvement. *p* = 0.307
Phase 2, effect of population information	Significant improvement, *p* = 0.047	Significant improvement, *p* = 0.007	No significant improvement. *p* = 0.177	No significant improvement. *p* = 0.233

* estimated difference between two samples.

** among participants who participated in both phases of the challenge.

### 3.3 Phase 1: TAC fitting using individual biokinetic data only

In this phase, each team received 5 time–activity points from 5 patients (patients 6–10) using a standardized time schedule (see Section 2.1). A total of 35 entries were submitted, with 33 fully complying with the challenge rules. Among the teams, 11 (31%) incorporated a data uncertainty model into their fit, while the rest did not or did not respond positively. Additionally, 4 (11%) performed only visual inspections, 5 (14%) used the AIC criterion, and 12 (34%) used RMSE as one of the checks of the goodness of fit. Only 6 (17%) teams considered population information. As for the objective functions, 15 (43%) used least squares, followed by 4 (11%) using nonlinear least squares.^20^ A variety of other objective functions were used by the other teams, as detailed in the Supporting Information (Appendix 4).

The ranking based on the RMSEs is shown in Figure 4; detailed results are provided in Appendix 5. The median (min, max) RMSE was 0.26 (0.16, 1.69), with the highest error, just like in phase 0 of the challenge, contributions coming from the blood (median RMSE = 0.15 (0.09, 1.57)). Kidneys and tumor had a relatively low median RMSE of 0.08 and 0.01, respectively. More details can be found in Table 3.

The best‐performing team, IRCCS‐Centro di Riferimento Oncologico di Aviano (see Appendix 3 for details), achieved a total RMSE score of 0.162, followed by the runner‐up with an RMSE of 0.184. Three groups had RMSE scores ranging from 0.19 to 0.195. Despite having the lowest total RMSE score, IRCCS ranked only 15th for tumor RMSE and 8th for blood RMSE.

As shown in Figure 4, only three of the top ten performing teams incorporated an uncertainty budget in their calculations. Our analysis found that teams using an uncertainty budget applied it consistently across all three organs in the challenge. However, the choice of objective function and fitting model varied by organ type, as detailed in Table 2. When comparing the performance of the 11 teams that used an uncertainty budget with those that did not, no statistically significant differences across any of the tissues analyzed were observed (Table 4).

### 3.4 Phase 2: TAC fitting with the incorporation of population‐based data

In the second competition phase, participants were provided standardized data points: five data points from five competition patients (patients 6–10, identical to Phase 1) and an additional 15 patients for population‐based estimates. A total of 28 entries fully complied with the challenge rules and were evaluated. Additionally, each team that participated in Phase 2 had already participated in Phase 1. A total of 10 teams (36%) incorporated a data uncertainty model into their fits; the rest either did not or did not provide a clear response. Despite phase 2 providing and encouraging population‐based information, only 13 teams (40%) utilized it. Additionally, 3 teams (11%) performed only visual inspection, 3 (11%) used the AIC criterion, and 11 (40%) used RMSE as one of their methods to check the goodness of fit. For the objective function, 12 teams (43%) used least squares, 8 (28%) used nonlinear least squares, and four other functions were used sporadically.

The best‐performing team in Phase 2, CCNM‐Cleveland Clinic (see Appendix 5 for details), achieved a total RMSE of 0.117. Their approach combined uncertainty balancing with available population‐based information. Organ‐level rankings showed CCNM placed third for kidney RMSE (0.040), first for blood RMSE (0.058), and 21st for tumor RMSE (0.020).

**FIGURE 5:**
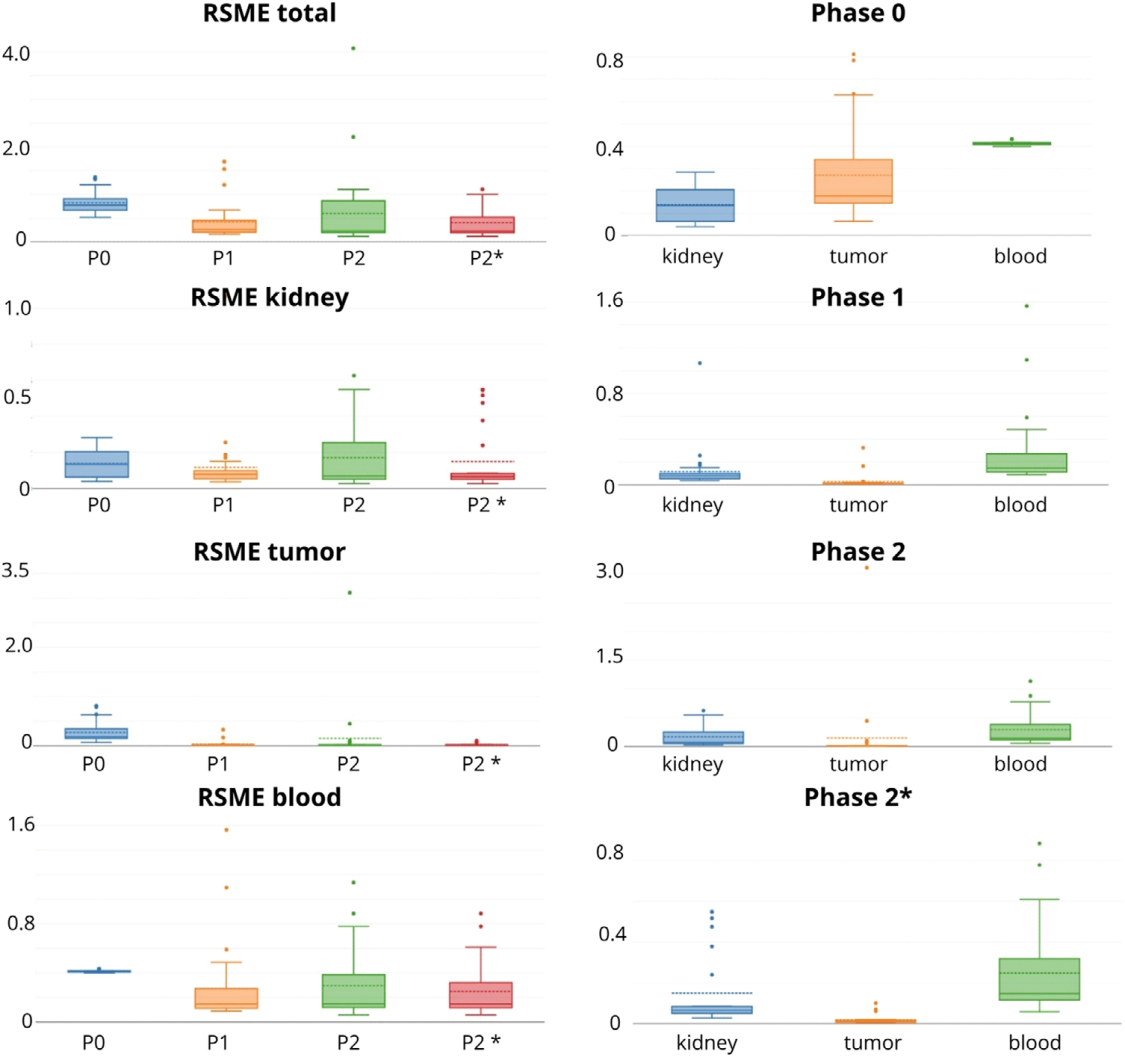
Plots visualizing changes in RMSE values (unit: h—kidney/blood and h/mL—tumor) throughout the challenge focus on average values as well as organ‐specific performance within and in‐between the phases of the challenge. (*excluding outlier teams).

Comparing teams that incorporated uncertainty in their TIAC calculations with those that did not reveal significant improvements in total RMSE (*p* = 0.037) and kidney RMSE (*p* = 0.026) when uncertainty was accounted for (Table 4, Figure 5).

## 4 DISCUSSION

In this report, we have summarized the main results of the TACTIC AAPM Grand Challenge 2023, the first large international competition focused on evaluating the factors that influence the accuracy of TIAC calculations for kidney, tumor, and blood tissues in [^177^Lu]Lu‐PSMA RPT. The challenge involved comparing TIAC values submitted by participants to ground truth values, which were synthetically calculated using the NLME^9^, ^11^, ^13^ model based on a large clinical dataset of [^177^Lu]Lu‐PSMA data. Through this challenge, we investigated the impact of key factors: the importance of an appropriate time schedule for post‐therapy biokinetic data,^16^ effect of late imaging time points, 4) the effect of an uncertainty budget,^15^ and the potential added value of population‐based kinetics in TIAC calculations.^4^, ^9^, ^10^, ^12^ The latter factors were found to significantly enhance the accuracy of the calculations.

From literature, it is well‐established that a well‐structured schedule for post‐therapy biokinetic data collection, particularly the inclusion of late time points, has a large effect on the accuracy and uncertainty of the calculated TIAC values. Our results from Phase 0 (Table 4) clearly demonstrated that the use of late time points (beyond 120 h) significantly improved the accuracy of the calculated TIAC for total RMSE, kidney, and blood measurements. However, this relationship did not hold for tumor kinetics. We anticipate that a similar relationship was present in the TACTIC patient population for all tissue types. Since the TIAC values for tumors were significantly lower compared to kidneys and blood (Appendix 1), it is likely that the 120‐h post‐injection time point was already relatively “functionally late” for tumors, thereby diminishing the observable impact of even later time points on tumor TIAC accuracy.

While designing the TACTIC challenge, our goal was to understand how the accuracy of TIAC calculations would evolve through the phases, providing participants with a steadily increasing amount of data and harmonization of the input data. The following anecdotal conclusions can be drawn:

1. As illustrated Figure 5, there was indeed a notable reduction in both the average RMSE across each tissue type and the total RMSE values from phase 0 to phase 1.
2. There was also no notable improvement observed in the total RMSE or in any of the three tissue types between phase 1 and phase 2.
3. As noted in Section 3.4, Phase 2 was the only phase of the challenge where we observed a significant improvement in the performance of teams that used uncertainty and/or population‐based information for the overall ranking, as well as for the kidney‐specific values. However, we did not find any significant performance improvement between the teams that utilized the uncertainty budget in both Phase 0 and Phase 1 of the challenge.

When focusing on the uncertainty budget, we did not observe any significant added value in its use for improving the accuracy of calculated TIACs across all tissue types in Phase 1. However, in Phase 2, a clear benefit of incorporating the uncertainty budget was evident for kidney and total RMSE values. Notably, 10 out of 33 eligible teams (33%) in Phase 1 were participating in the challenge for the first time. Phase 2 analysis clearly demonstrated the added value of uncertainty calculations for two tissue types, which aligns with the existing literature. This improvement was observed despite the wide variety of fitting methods and objective functions used by participants in the TACTIC challenge. For tumor and blood TIAC values within Phase 2, the added value of uncertainty calculations was not evident.

Increasingly, studies highlight the potential added value of population‐based kinetic modeling for RPT dosimetry applications.^9^, ^10^, ^12^ In Phase 2 of the challenge, where data from 15 additional patients with similar tracer kinetics was provided, we anticipated better RMSE performance from teams utilizing this information as a population‐based input for calculating patient‐specific TIACs. For kidney TIACs, we observed a benefit from using population‐based information, despite the varied fitting models and objective functions employed by participants. However, for tumor and blood TIACs, the use of population‐based kinetics did not result in improved performance. We hypothesize that, unlike organs at risk, tumors and blood may exhibit greater variability in biokinetics both across different patients as within a single patient (though this was not investigated here). As a result, population‐based kinetic modeling alone may not offer as strong an improvement for tumors as it does for organs at risk. Therefore, further investigation into multi‐parametric modeling and the evaluation of additional biodata is important to enhance tumor dosimetry through population‐based kinetic modeling.^4^ Alternatively, the evaluation of population‐based effects on the tumor and blood may have been impacted by the higher uncertainty in activity calculations for these organs (i.e., related to dispersion). This increased uncertainty could necessitate greater statistical power to detect effects of various techniques, which we may not have achieved in phase 2. A similar rationale may be valid to the effect of uncertainty on the accuracy of calculated TIACs for blood and tumor as well.

Finally, we would like to highlight some limitations from the design and analysis of the challenge. The challenge was initially designed with equal weighting of RMSE values for the three tissue types (i.e., kidney, tumor, and blood). However, throughout the challenge, we observed that these organs contribute unequally to the total RMSE value (Figure 5, Table 4), with blood consistently showing the largest error, followed by the kidney and tumor. This design unintentionally introduced bias into the final ranking, making it difficult for teams with the best‐performing tumor TIACs—arguably the most critical organ for assessing a patient's response and potential absorbed dose‐effects—to win a phase of the challenge. Therefore, we believe it is important to introduce organ‐based weighting in the total RMSE calculation or define a winner per tissue type, if a similar challenge is conducted in the future.

Additionally, our goal to evaluate the effects of various fit and objective functions on the accuracy of calculated TIACs was hindered by the wide range of functions, although of similar type, used by participants with a relatively small dataset, making meaningful statistical comparisons difficult. The diversity of functions employed to fit biokinetic data for the same tissue using identical datasets was unexpected. In each phase, up to 15 different fit and/or objective function variations were used, highlighting a lack of consensus on the best approach.

We also noted significant inter‐team variability within each phase, though this variability decreased with the introduction of uncertainty budget calculations. Despite this, the variability remained considerable. As post‐therapy dosimetry becomes a legal requirement in many European countries (European Directive 2013/59/Euratom), this ensures that consistent quality of care and accurate absorbed dose‐effect calculations across hospitals is critical.^21^ Given the high inter‐team variability in TIAC calculation methodologies and resulting values, it is essential to establish better guidelines and harmonization efforts to improve reproducibility and comparability. This is particularly important for tumor kinetics, which are crucial for absorbed dose‐effect assessments and where we observed the greatest variation and an unexpected lack of benefit from incorporating an uncertainty budget. Significant education and standardization efforts are needed to support large‐scale studies that will enhance our understanding and personalization of [^177^Lu]Lu‐PSMA therapy.

We believe that the TACTIC AAPM Grand Challenge has clearly illustrated the importance of various factors in time‐activity curve fitting for RPT applications, including the significance of education, late time‐point imaging, the use of an uncertainty budget, and the potential of population‐based kinetic modeling. This was evident even with a dataset consisting of a mix of fit functions and model selection approaches. We are confident that the findings and conclusions described in this manuscript can serve as a foundation for educational resources and the design of standardization and harmonization practices, the need for which was clearly highlighted by our results.

## 5 CONCLUSION

The TACTIC AAPM Grand Challenge was an international effort to evaluate the effects of time schedule, uncertainty balance, and population‐based information on the accuracy of TIAC calculations for three tissue types in [^177^]Lu]Lu‐PSMA therapy, using synthetically generated data. The challenge highlighted that incorporating an uncertainty budget and population‐based information improved TIAC calculations for kidneys, but only when participants had sufficient experience with the data and an adequate patient population. These findings underscore the potential for improving dosimetry accuracy through population‐based kinetic models and uncertainty‐budget‐driven model selection, while also emphasizing the need for guidance on effectively incorporating these aspects into the RPT dosimetry workflow.

## CONFLICT OF INTEREST STATEMENT

JOD is an employee of Siemens Medical Solutions USA, who did not finance or sponsor this work. G.G. and D.H. serve as consultants for ITM Oncologics GmbH, Garching, Germany. The remaining authors declare no conflicts of interest.

## Supporting information



Supporting Information

## Data Availability

The TACTIC challenge data are available at the challenge website: https://www.aapm.org/GrandChallenge/TACTIC/default.asp. Data is also available from the corresponding author upon reasonable request.
