# Supplementary material for: Understanding time–activity curve and time‐integrated activity variations in radiopharmaceutical therapy challenge: Experience and results
Source: Med Phys. 2025 Sep 26;52(10):e70043. doi: 10.1002/mp.70043 (PMC12464796; doi:10.1002/mp.70043)
Supplement: Supplementary file 1 — Supporting Information [file MP-52-0-s001.doc]

**SUPPORTING INFORMATION FILE**

**Understanding Time-Activity Curve and Time-Integrated Activity Variations in Radiopharmaceutical Therapy Challenge: experience and results**

Oleksandra V. Ivashchenko*^§^ ^1^, Jim O'Doherty^§ 2,3,4^, Deni Hardiansyah ^5^, Elisa Grassi ^6^, Johannes Tran-Gia ^7^, Johannes W.T. Heemskerk ^8^, Eero Hippeläinen ^9^, Mattias Sandström ^10^, Marta Cremonesi ^11^, Gerhard Glatting ^12^.

1. Department of Nuclear Medicine and Molecular Imaging, University Medical Center Groningen, 9713GZ, Groningen, The Netherlands
2. R&D Collaborations, Siemens Medical Solutions, Malvern, PA 19355, USA
3. Department of Radiology and Radiological Science, Medical University of South Carolina, Charleston, SC 29425, USA
4. Department of Radiography & Diagnostic Imaging, University College Dublin, Dublin, D04 V1W8, Ireland
5. Department of Physics, FMIPA, Universitas Indonesia, Depok, 16424, Indonesia
6. Medical Physics Unit, Azienda USL-IRCCS di Reggio Emilia, Reggio Emilia, 42122, Italy
7. Department of Nuclear Medicine, University Hospital Würzburg, Würzburg, 97080, Germany
8. Department of Radiology, Leiden University Medical Center, Leiden, South Holland, 2333 ZG, The Netherlands
9. Department of Physics, University of Helsinki and Helsinki University Hospital, Helsinki, 00014, Finland
10. Department of Radiology, Uppsala University, Uppsala, 75105, Sweden
11. Radiation Research Unit, European Institute of Oncology, Milan, 20241, Italy
12. Department of Nuclear Medicine, Ulm University, Ulm, 89081, Germany

§ equal contribution

*** Corresponding author:**

O.V. Ivashchenko, PhD

University Medical Center Groningen

Department of Nuclear Medicine and Molecular Imaging

Hanzeplein 1, 9713GZ, the Netherlands

Email: [o.v.ivashchenko@umcg.nl](mailto:o.v.ivashchenko@umcg.nl)

Appendix 1: Kinetic Data used for the TACTIC challenge

**Table S1**. The kinetic data of five test patients used in Phase 0 of the challenge were sampled on a 1-hour grid using the NLME model described in Section 2.1. This approach allowed for custom data point selection during the warm-up phase of the challenge. A lookup table was created based on the time points requested by the participants, enabling the distribution of the corresponding data to them.

| **Hours p.i.** | **% Administered Activity** | | | | | **%Administered Activity/Volume** | | | | | **% Administered Activity** | | | | |
| --- | --- | --- | --- | --- | --- | --- | --- | --- | --- | --- | --- | --- | --- | --- | --- |
|  |  |  |  |  |  |  |  |  |  |  |  |  |  |  |  |
|  | **Kidneys** | | | | | **Tumor** | | | | | **Blood** | | | | |
|  | **P1** | **P2** | **P3** | **P4** | **P5** | **P1** | **P2** | **P3** | **P4** | **P5** | **P1** | **P2** | **P3** | **P4** | **P5** |
| 1 | 0.853 | 1.937 | 3.618 | 1.637 | 0.798 | 0.029 | 0.026 | 0.012 | 0.013 | 0.010 | 12.199 | 7.345 | 14.523 | 20.355 | 20.354 |
| 2 | 1.110 | 1.722 | 3.173 | 1.564 | 0.678 | 0.023 | 0.022 | 0.012 | 0.015 | 0.011 | 9.203 | 4.539 | 11.955 | 16.754 | 13.622 |
| 3 | 0.851 | 1.806 | 3.208 | 1.792 | 0.634 | 0.030 | 0.028 | 0.015 | 0.014 | 0.010 | 7.046 | 2.580 | 8.038 | 14.030 | 10.654 |
| 4 | 0.973 | 1.402 | 2.975 | 1.650 | 0.615 | 0.028 | 0.023 | 0.013 | 0.011 | 0.014 | 4.828 | 1.816 | 6.683 | 12.703 | 9.095 |
| 5 | 1.101 | 1.366 | 3.290 | 1.731 | 0.689 | 0.028 | 0.025 | 0.011 | 0.015 | 0.011 | 3.826 | 1.543 | 5.459 | 11.180 | 8.590 |
| 6 | 0.942 | 1.156 | 3.024 | 1.699 | 0.559 | 0.031 | 0.022 | 0.013 | 0.014 | 0.012 | 3.284 | 1.143 | 4.953 | 10.147 | 6.912 |
| 7 | 0.906 | 1.164 | 3.064 | 1.403 | 0.636 | 0.043 | 0.027 | 0.012 | 0.012 | 0.012 | 2.569 | 1.003 | 4.144 | 9.332 | 6.541 |
| 8 | 0.984 | 0.941 | 2.498 | 1.534 | 0.664 | 0.044 | 0.017 | 0.012 | 0.012 | 0.014 | 2.311 | 0.818 | 3.068 | 7.559 | 5.015 |
| 9 | 1.056 | 1.167 | 2.847 | 1.706 | 0.557 | 0.033 | 0.029 | 0.013 | 0.010 | 0.010 | 1.919 | 0.715 | 3.473 | 8.982 | 4.818 |
| 10 | 1.036 | 0.954 | 2.766 | 1.279 | 0.607 | 0.031 | 0.021 | 0.016 | 0.011 | 0.014 | 1.892 | 0.713 | 2.763 | 6.360 | 3.698 |
| 11 | 0.986 | 0.835 | 2.529 | 1.478 | 0.589 | 0.043 | 0.034 | 0.018 | 0.010 | 0.014 | 1.972 | 0.636 | 2.660 | 6.297 | 3.942 |
| 12 | 0.976 | 0.774 | 2.573 | 1.611 | 0.575 | 0.049 | 0.026 | 0.011 | 0.011 | 0.014 | 1.722 | 0.630 | 2.748 | 5.643 | 3.608 |
| 13 | 1.068 | 0.709 | 2.567 | 1.260 | 0.533 | 0.043 | 0.030 | 0.017 | 0.010 | 0.013 | 1.475 | 0.631 | 2.277 | 5.073 | 3.535 |
| 14 | 0.841 | 0.673 | 2.332 | 1.253 | 0.478 | 0.041 | 0.020 | 0.016 | 0.010 | 0.017 | 1.349 | 0.635 | 2.172 | 5.060 | 3.242 |
| 15 | 1.001 | 0.655 | 2.172 | 1.596 | 0.469 | 0.041 | 0.028 | 0.016 | 0.009 | 0.013 | 1.259 | 0.539 | 2.279 | 5.288 | 3.349 |
| 16 | 0.887 | 0.580 | 2.288 | 1.353 | 0.521 | 0.045 | 0.022 | 0.018 | 0.012 | 0.015 | 1.233 | 0.519 | 1.829 | 4.610 | 2.513 |
| 17 | 0.881 | 0.459 | 2.034 | 1.543 | 0.542 | 0.041 | 0.031 | 0.018 | 0.009 | 0.012 | 1.135 | 0.540 | 1.662 | 4.504 | 2.738 |
| 18 | 0.822 | 0.550 | 2.207 | 1.402 | 0.449 | 0.053 | 0.022 | 0.019 | 0.010 | 0.012 | 1.051 | 0.498 | 1.603 | 4.020 | 2.666 |
| 19 | 0.820 | 0.540 | 1.744 | 1.452 | 0.595 | 0.038 | 0.027 | 0.018 | 0.008 | 0.015 | 0.982 | 0.495 | 1.631 | 3.497 | 2.137 |
| 20 | 1.027 | 0.474 | 2.037 | 1.556 | 0.500 | 0.048 | 0.019 | 0.019 | 0.013 | 0.017 | 1.051 | 0.461 | 1.432 | 3.522 | 2.227 |
| 21 | 0.852 | 0.434 | 1.916 | 1.293 | 0.510 | 0.042 | 0.018 | 0.015 | 0.008 | 0.015 | 0.838 | 0.448 | 1.692 | 3.150 | 1.769 |
| 22 | 0.828 | 0.510 | 2.082 | 1.261 | 0.562 | 0.050 | 0.016 | 0.018 | 0.009 | 0.014 | 0.690 | 0.462 | 1.328 | 2.838 | 1.865 |
| 23 | 0.950 | 0.422 | 1.901 | 1.262 | 0.512 | 0.038 | 0.029 | 0.018 | 0.009 | 0.014 | 0.783 | 0.416 | 1.361 | 2.859 | 1.732 |
| 24 | 0.854 | 0.377 | 1.562 | 1.146 | 0.461 | 0.035 | 0.034 | 0.015 | 0.009 | 0.018 | 0.641 | 0.379 | 1.245 | 2.428 | 1.579 |
| 25 | 1.046 | 0.431 | 1.708 | 1.266 | 0.559 | 0.051 | 0.026 | 0.018 | 0.007 | 0.014 | 0.655 | 0.404 | 1.200 | 2.408 | 1.433 |
| 26 | 0.949 | 0.378 | 1.718 | 1.353 | 0.492 | 0.029 | 0.018 | 0.015 | 0.008 | 0.017 | 0.633 | 0.423 | 1.066 | 2.245 | 1.459 |
| 27 | 0.915 | 0.429 | 1.693 | 1.291 | 0.471 | 0.038 | 0.023 | 0.018 | 0.008 | 0.013 | 0.567 | 0.377 | 1.138 | 2.192 | 1.391 |
| 28 | 0.919 | 0.358 | 1.541 | 1.075 | 0.439 | 0.041 | 0.026 | 0.018 | 0.009 | 0.017 | 0.551 | 0.398 | 0.867 | 2.152 | 1.485 |
| 29 | 0.883 | 0.360 | 1.511 | 1.157 | 0.568 | 0.048 | 0.017 | 0.019 | 0.007 | 0.016 | 0.455 | 0.343 | 1.035 | 2.033 | 1.256 |
| 30 | 0.752 | 0.320 | 1.457 | 1.217 | 0.499 | 0.040 | 0.019 | 0.018 | 0.009 | 0.009 | 0.506 | 0.339 | 0.848 | 1.733 | 1.401 |
| 31 | 0.756 | 0.417 | 1.406 | 1.198 | 0.563 | 0.036 | 0.025 | 0.014 | 0.006 | 0.016 | 0.445 | 0.397 | 0.806 | 1.873 | 1.105 |
| 32 | 0.709 | 0.379 | 1.420 | 1.121 | 0.504 | 0.042 | 0.025 | 0.018 | 0.005 | 0.013 | 0.448 | 0.385 | 0.816 | 1.581 | 1.097 |
| 33 | 0.756 | 0.365 | 1.362 | 1.185 | 0.498 | 0.048 | 0.027 | 0.016 | 0.006 | 0.020 | 0.431 | 0.343 | 0.662 | 1.301 | 1.102 |
| 34 | 0.747 | 0.382 | 1.444 | 1.174 | 0.460 | 0.037 | 0.036 | 0.018 | 0.006 | 0.019 | 0.370 | 0.290 | 0.767 | 1.559 | 0.961 |
| 35 | 0.697 | 0.337 | 1.397 | 1.155 | 0.520 | 0.050 | 0.020 | 0.016 | 0.005 | 0.013 | 0.345 | 0.346 | 0.674 | 1.327 | 0.896 |
| 36 | 0.749 | 0.379 | 1.376 | 1.069 | 0.456 | 0.054 | 0.022 | 0.014 | 0.008 | 0.015 | 0.372 | 0.353 | 0.608 | 1.334 | 0.870 |
| 37 | 0.814 | 0.344 | 1.372 | 1.130 | 0.470 | 0.042 | 0.028 | 0.018 | 0.006 | 0.011 | 0.324 | 0.349 | 0.646 | 1.183 | 0.894 |
| 38 | 0.744 | 0.391 | 1.278 | 1.009 | 0.536 | 0.040 | 0.028 | 0.017 | 0.007 | 0.014 | 0.314 | 0.327 | 0.573 | 1.147 | 0.827 |
| 39 | 0.604 | 0.394 | 1.346 | 1.075 | 0.580 | 0.037 | 0.018 | 0.015 | 0.005 | 0.017 | 0.300 | 0.318 | 0.524 | 1.115 | 0.786 |
| 40 | 0.660 | 0.353 | 1.283 | 1.012 | 0.507 | 0.039 | 0.025 | 0.014 | 0.004 | 0.017 | 0.286 | 0.344 | 0.601 | 1.047 | 0.645 |
| 41 | 0.676 | 0.338 | 1.134 | 0.984 | 0.470 | 0.044 | 0.026 | 0.014 | 0.006 | 0.016 | 0.261 | 0.305 | 0.504 | 0.924 | 0.544 |
| 42 | 0.568 | 0.343 | 1.197 | 0.996 | 0.463 | 0.028 | 0.027 | 0.014 | 0.006 | 0.020 | 0.274 | 0.299 | 0.493 | 0.952 | 0.645 |
| 43 | 0.556 | 0.293 | 1.253 | 0.743 | 0.409 | 0.038 | 0.026 | 0.018 | 0.006 | 0.015 | 0.266 | 0.283 | 0.439 | 0.969 | 0.585 |
| 44 | 0.585 | 0.315 | 1.019 | 0.865 | 0.497 | 0.050 | 0.017 | 0.013 | 0.005 | 0.013 | 0.243 | 0.277 | 0.373 | 0.907 | 0.573 |
| 45 | 0.578 | 0.289 | 1.242 | 0.964 | 0.467 | 0.040 | 0.026 | 0.017 | 0.005 | 0.016 | 0.234 | 0.277 | 0.416 | 0.812 | 0.550 |
| 46 | 0.630 | 0.303 | 0.990 | 0.926 | 0.483 | 0.036 | 0.021 | 0.015 | 0.004 | 0.019 | 0.243 | 0.292 | 0.423 | 0.868 | 0.474 |
| 47 | 0.596 | 0.342 | 1.145 | 0.871 | 0.511 | 0.049 | 0.022 | 0.016 | 0.005 | 0.016 | 0.236 | 0.276 | 0.387 | 0.628 | 0.481 |
| 48 | 0.631 | 0.315 | 1.109 | 0.925 | 0.484 | 0.041 | 0.021 | 0.012 | 0.005 | 0.015 | 0.207 | 0.274 | 0.367 | 0.731 | 0.505 |
| 49 | 0.595 | 0.289 | 1.044 | 0.947 | 0.443 | 0.031 | 0.028 | 0.017 | 0.004 | 0.011 | 0.216 | 0.246 | 0.311 | 0.805 | 0.475 |
| 50 | 0.559 | 0.347 | 1.170 | 0.861 | 0.371 | 0.031 | 0.022 | 0.013 | 0.004 | 0.014 | 0.223 | 0.264 | 0.389 | 0.637 | 0.456 |
| 51 | 0.609 | 0.326 | 0.979 | 0.770 | 0.404 | 0.045 | 0.020 | 0.018 | 0.004 | 0.013 | 0.181 | 0.284 | 0.333 | 0.696 | 0.430 |
| 52 | 0.554 | 0.351 | 1.100 | 0.818 | 0.466 | 0.044 | 0.017 | 0.016 | 0.004 | 0.011 | 0.180 | 0.253 | 0.333 | 0.598 | 0.330 |
| 53 | 0.490 | 0.282 | 1.092 | 0.815 | 0.481 | 0.039 | 0.018 | 0.015 | 0.004 | 0.015 | 0.164 | 0.290 | 0.300 | 0.527 | 0.372 |
| 54 | 0.570 | 0.269 | 1.068 | 0.939 | 0.442 | 0.041 | 0.027 | 0.015 | 0.004 | 0.009 | 0.160 | 0.283 | 0.301 | 0.588 | 0.380 |
| 55 | 0.549 | 0.293 | 0.939 | 0.641 | 0.449 | 0.034 | 0.019 | 0.012 | 0.004 | 0.012 | 0.174 | 0.265 | 0.298 | 0.545 | 0.378 |
| 56 | 0.507 | 0.264 | 0.970 | 0.703 | 0.357 | 0.034 | 0.021 | 0.017 | 0.004 | 0.016 | 0.144 | 0.240 | 0.329 | 0.463 | 0.302 |
| 57 | 0.498 | 0.268 | 1.063 | 0.857 | 0.453 | 0.029 | 0.023 | 0.015 | 0.005 | 0.014 | 0.154 | 0.262 | 0.311 | 0.487 | 0.323 |
| 58 | 0.457 | 0.275 | 0.917 | 0.764 | 0.476 | 0.034 | 0.030 | 0.013 | 0.004 | 0.011 | 0.164 | 0.254 | 0.289 | 0.426 | 0.344 |
| 59 | 0.508 | 0.287 | 1.036 | 0.724 | 0.445 | 0.035 | 0.025 | 0.012 | 0.003 | 0.014 | 0.142 | 0.218 | 0.276 | 0.438 | 0.277 |
| 60 | 0.432 | 0.304 | 0.899 | 0.686 | 0.412 | 0.021 | 0.027 | 0.015 | 0.004 | 0.014 | 0.155 | 0.242 | 0.263 | 0.376 | 0.290 |
| 61 | 0.415 | 0.321 | 0.940 | 0.653 | 0.449 | 0.021 | 0.022 | 0.012 | 0.004 | 0.015 | 0.159 | 0.253 | 0.280 | 0.418 | 0.279 |
| 62 | 0.456 | 0.309 | 0.826 | 0.704 | 0.376 | 0.033 | 0.013 | 0.012 | 0.005 | 0.012 | 0.125 | 0.225 | 0.262 | 0.392 | 0.242 |
| 63 | 0.409 | 0.300 | 0.823 | 0.634 | 0.399 | 0.031 | 0.021 | 0.012 | 0.004 | 0.011 | 0.117 | 0.248 | 0.240 | 0.327 | 0.259 |
| 64 | 0.441 | 0.284 | 0.862 | 0.652 | 0.396 | 0.039 | 0.024 | 0.013 | 0.003 | 0.014 | 0.130 | 0.252 | 0.233 | 0.362 | 0.261 |
| 65 | 0.477 | 0.228 | 0.790 | 0.663 | 0.399 | 0.029 | 0.024 | 0.011 | 0.003 | 0.016 | 0.115 | 0.222 | 0.225 | 0.364 | 0.252 |
| 66 | 0.449 | 0.260 | 0.869 | 0.630 | 0.391 | 0.034 | 0.019 | 0.015 | 0.003 | 0.015 | 0.106 | 0.253 | 0.203 | 0.309 | 0.236 |
| 67 | 0.379 | 0.301 | 0.795 | 0.626 | 0.390 | 0.033 | 0.018 | 0.017 | 0.003 | 0.012 | 0.118 | 0.242 | 0.217 | 0.297 | 0.249 |
| 68 | 0.401 | 0.281 | 0.800 | 0.619 | 0.348 | 0.028 | 0.014 | 0.009 | 0.003 | 0.012 | 0.121 | 0.237 | 0.237 | 0.322 | 0.194 |
| 69 | 0.353 | 0.277 | 0.734 | 0.613 | 0.368 | 0.028 | 0.018 | 0.014 | 0.003 | 0.011 | 0.126 | 0.254 | 0.220 | 0.299 | 0.216 |
| 70 | 0.430 | 0.275 | 0.835 | 0.673 | 0.324 | 0.034 | 0.013 | 0.012 | 0.003 | 0.011 | 0.111 | 0.256 | 0.216 | 0.267 | 0.211 |
| 71 | 0.353 | 0.259 | 0.885 | 0.561 | 0.369 | 0.030 | 0.017 | 0.008 | 0.003 | 0.013 | 0.102 | 0.252 | 0.205 | 0.231 | 0.215 |
| 72 | 0.429 | 0.271 | 0.758 | 0.588 | 0.334 | 0.027 | 0.018 | 0.011 | 0.003 | 0.013 | 0.123 | 0.268 | 0.210 | 0.262 | 0.217 |
| 73 | 0.312 | 0.306 | 0.678 | 0.677 | 0.353 | 0.035 | 0.012 | 0.017 | 0.003 | 0.013 | 0.124 | 0.251 | 0.185 | 0.243 | 0.210 |
| 74 | 0.338 | 0.244 | 0.712 | 0.566 | 0.336 | 0.022 | 0.020 | 0.013 | 0.003 | 0.013 | 0.112 | 0.246 | 0.191 | 0.241 | 0.197 |
| 75 | 0.348 | 0.244 | 0.685 | 0.595 | 0.360 | 0.027 | 0.017 | 0.012 | 0.003 | 0.012 | 0.111 | 0.219 | 0.195 | 0.266 | 0.192 |
| 76 | 0.373 | 0.255 | 0.800 | 0.592 | 0.294 | 0.031 | 0.017 | 0.012 | 0.003 | 0.012 | 0.086 | 0.230 | 0.186 | 0.222 | 0.193 |
| 77 | 0.392 | 0.256 | 0.757 | 0.576 | 0.340 | 0.029 | 0.020 | 0.015 | 0.003 | 0.012 | 0.098 | 0.228 | 0.171 | 0.218 | 0.185 |
| 78 | 0.350 | 0.270 | 0.787 | 0.506 | 0.337 | 0.023 | 0.016 | 0.014 | 0.003 | 0.010 | 0.103 | 0.250 | 0.198 | 0.190 | 0.191 |
| 79 | 0.329 | 0.252 | 0.706 | 0.604 | 0.342 | 0.028 | 0.017 | 0.011 | 0.003 | 0.010 | 0.099 | 0.216 | 0.176 | 0.187 | 0.186 |
| 80 | 0.292 | 0.192 | 0.750 | 0.549 | 0.324 | 0.029 | 0.019 | 0.014 | 0.003 | 0.012 | 0.102 | 0.218 | 0.169 | 0.179 | 0.162 |
| 81 | 0.280 | 0.248 | 0.636 | 0.516 | 0.335 | 0.017 | 0.021 | 0.015 | 0.003 | 0.011 | 0.095 | 0.238 | 0.195 | 0.186 | 0.171 |
| 82 | 0.313 | 0.288 | 0.650 | 0.509 | 0.339 | 0.027 | 0.016 | 0.012 | 0.002 | 0.011 | 0.090 | 0.206 | 0.168 | 0.187 | 0.166 |
| 83 | 0.328 | 0.231 | 0.583 | 0.577 | 0.319 | 0.029 | 0.018 | 0.011 | 0.003 | 0.010 | 0.090 | 0.223 | 0.169 | 0.161 | 0.166 |
| 84 | 0.330 | 0.248 | 0.654 | 0.509 | 0.291 | 0.029 | 0.012 | 0.010 | 0.003 | 0.012 | 0.093 | 0.233 | 0.165 | 0.153 | 0.154 |
| 85 | 0.296 | 0.246 | 0.617 | 0.558 | 0.307 | 0.025 | 0.021 | 0.013 | 0.002 | 0.014 | 0.084 | 0.234 | 0.152 | 0.171 | 0.150 |
| 86 | 0.322 | 0.243 | 0.654 | 0.444 | 0.290 | 0.026 | 0.015 | 0.012 | 0.002 | 0.010 | 0.088 | 0.235 | 0.162 | 0.179 | 0.153 |
| 87 | 0.319 | 0.263 | 0.580 | 0.499 | 0.307 | 0.023 | 0.018 | 0.011 | 0.003 | 0.014 | 0.090 | 0.208 | 0.141 | 0.155 | 0.142 |
| 88 | 0.302 | 0.232 | 0.563 | 0.534 | 0.309 | 0.022 | 0.011 | 0.011 | 0.002 | 0.014 | 0.080 | 0.216 | 0.152 | 0.134 | 0.136 |
| 89 | 0.292 | 0.246 | 0.672 | 0.509 | 0.271 | 0.029 | 0.015 | 0.014 | 0.002 | 0.009 | 0.091 | 0.219 | 0.158 | 0.126 | 0.142 |
| 90 | 0.271 | 0.236 | 0.656 | 0.473 | 0.296 | 0.015 | 0.012 | 0.012 | 0.002 | 0.012 | 0.085 | 0.225 | 0.139 | 0.142 | 0.125 |
| 91 | 0.288 | 0.205 | 0.515 | 0.401 | 0.307 | 0.023 | 0.016 | 0.013 | 0.002 | 0.011 | 0.091 | 0.177 | 0.123 | 0.134 | 0.124 |
| 92 | 0.240 | 0.277 | 0.583 | 0.417 | 0.288 | 0.026 | 0.022 | 0.010 | 0.003 | 0.009 | 0.088 | 0.212 | 0.158 | 0.139 | 0.127 |
| 93 | 0.276 | 0.217 | 0.578 | 0.445 | 0.264 | 0.026 | 0.016 | 0.009 | 0.002 | 0.010 | 0.079 | 0.194 | 0.153 | 0.114 | 0.140 |
| 94 | 0.309 | 0.235 | 0.530 | 0.444 | 0.251 | 0.025 | 0.018 | 0.011 | 0.002 | 0.012 | 0.075 | 0.209 | 0.122 | 0.125 | 0.137 |
| 95 | 0.221 | 0.227 | 0.536 | 0.412 | 0.229 | 0.019 | 0.016 | 0.011 | 0.002 | 0.009 | 0.072 | 0.188 | 0.147 | 0.142 | 0.120 |
| 96 | 0.226 | 0.217 | 0.550 | 0.435 | 0.239 | 0.018 | 0.017 | 0.011 | 0.001 | 0.010 | 0.079 | 0.182 | 0.136 | 0.132 | 0.118 |
| 97 | 0.263 | 0.222 | 0.583 | 0.440 | 0.247 | 0.021 | 0.015 | 0.008 | 0.001 | 0.011 | 0.090 | 0.193 | 0.130 | 0.112 | 0.116 |
| 98 | 0.284 | 0.202 | 0.498 | 0.457 | 0.265 | 0.019 | 0.015 | 0.010 | 0.002 | 0.012 | 0.074 | 0.189 | 0.144 | 0.118 | 0.136 |
| 99 | 0.252 | 0.241 | 0.564 | 0.356 | 0.259 | 0.022 | 0.015 | 0.011 | 0.002 | 0.011 | 0.073 | 0.193 | 0.125 | 0.100 | 0.113 |
| 100 | 0.259 | 0.235 | 0.508 | 0.463 | 0.234 | 0.018 | 0.015 | 0.007 | 0.002 | 0.010 | 0.080 | 0.222 | 0.137 | 0.110 | 0.111 |
| 101 | 0.269 | 0.167 | 0.525 | 0.325 | 0.249 | 0.021 | 0.014 | 0.008 | 0.002 | 0.013 | 0.075 | 0.216 | 0.121 | 0.105 | 0.121 |
| 102 | 0.256 | 0.196 | 0.501 | 0.450 | 0.245 | 0.028 | 0.015 | 0.010 | 0.002 | 0.009 | 0.075 | 0.189 | 0.124 | 0.112 | 0.105 |
| 103 | 0.240 | 0.208 | 0.531 | 0.387 | 0.217 | 0.021 | 0.014 | 0.012 | 0.001 | 0.012 | 0.067 | 0.198 | 0.130 | 0.101 | 0.112 |
| 104 | 0.236 | 0.209 | 0.524 | 0.315 | 0.289 | 0.017 | 0.013 | 0.008 | 0.002 | 0.012 | 0.065 | 0.190 | 0.115 | 0.093 | 0.107 |
| 105 | 0.231 | 0.196 | 0.531 | 0.409 | 0.235 | 0.018 | 0.018 | 0.008 | 0.001 | 0.010 | 0.077 | 0.204 | 0.101 | 0.102 | 0.105 |
| 106 | 0.260 | 0.191 | 0.489 | 0.333 | 0.228 | 0.020 | 0.016 | 0.007 | 0.001 | 0.009 | 0.064 | 0.207 | 0.115 | 0.100 | 0.092 |
| 107 | 0.230 | 0.178 | 0.456 | 0.333 | 0.213 | 0.019 | 0.011 | 0.008 | 0.002 | 0.010 | 0.067 | 0.191 | 0.110 | 0.100 | 0.102 |
| 108 | 0.222 | 0.197 | 0.467 | 0.370 | 0.230 | 0.021 | 0.008 | 0.010 | 0.001 | 0.012 | 0.064 | 0.191 | 0.083 | 0.084 | 0.103 |
| 109 | 0.238 | 0.183 | 0.461 | 0.346 | 0.225 | 0.020 | 0.016 | 0.008 | 0.001 | 0.010 | 0.065 | 0.177 | 0.115 | 0.099 | 0.094 |
| 110 | 0.193 | 0.167 | 0.456 | 0.338 | 0.239 | 0.017 | 0.015 | 0.007 | 0.001 | 0.010 | 0.067 | 0.183 | 0.097 | 0.080 | 0.088 |
| 111 | 0.229 | 0.198 | 0.471 | 0.302 | 0.220 | 0.021 | 0.011 | 0.009 | 0.002 | 0.011 | 0.063 | 0.194 | 0.096 | 0.089 | 0.089 |
| 112 | 0.245 | 0.210 | 0.416 | 0.296 | 0.199 | 0.021 | 0.014 | 0.008 | 0.001 | 0.010 | 0.061 | 0.198 | 0.101 | 0.086 | 0.096 |
| 113 | 0.194 | 0.166 | 0.395 | 0.317 | 0.207 | 0.018 | 0.013 | 0.008 | 0.001 | 0.010 | 0.066 | 0.172 | 0.099 | 0.085 | 0.098 |
| 114 | 0.224 | 0.189 | 0.476 | 0.268 | 0.200 | 0.016 | 0.014 | 0.009 | 0.001 | 0.011 | 0.058 | 0.167 | 0.098 | 0.086 | 0.094 |
| 115 | 0.204 | 0.171 | 0.433 | 0.352 | 0.204 | 0.016 | 0.012 | 0.006 | 0.002 | 0.007 | 0.060 | 0.199 | 0.115 | 0.087 | 0.096 |
| 116 | 0.212 | 0.157 | 0.427 | 0.328 | 0.191 | 0.020 | 0.018 | 0.009 | 0.002 | 0.009 | 0.054 | 0.198 | 0.103 | 0.077 | 0.086 |
| 117 | 0.200 | 0.146 | 0.476 | 0.253 | 0.184 | 0.018 | 0.016 | 0.007 | 0.001 | 0.011 | 0.063 | 0.187 | 0.096 | 0.061 | 0.081 |
| 118 | 0.166 | 0.185 | 0.408 | 0.308 | 0.207 | 0.019 | 0.011 | 0.009 | 0.001 | 0.010 | 0.062 | 0.181 | 0.093 | 0.060 | 0.071 |
| 119 | 0.212 | 0.178 | 0.357 | 0.285 | 0.194 | 0.017 | 0.014 | 0.007 | 0.001 | 0.010 | 0.058 | 0.172 | 0.095 | 0.071 | 0.094 |
| 120 | 0.195 | 0.175 | 0.371 | 0.336 | 0.167 | 0.013 | 0.009 | 0.006 | 0.001 | 0.011 | 0.057 | 0.168 | 0.095 | 0.072 | 0.071 |
| 121 | 0.201 | 0.150 | 0.413 | 0.319 | 0.177 | 0.016 | 0.011 | 0.007 | 0.001 | 0.009 | 0.055 | 0.188 | 0.095 | 0.078 | 0.078 |
| 122 | 0.210 | 0.175 | 0.450 | 0.291 | 0.172 | 0.013 | 0.013 | 0.009 | 0.001 | 0.010 | 0.052 | 0.168 | 0.096 | 0.074 | 0.069 |
| 123 | 0.200 | 0.152 | 0.375 | 0.295 | 0.153 | 0.015 | 0.012 | 0.005 | 0.001 | 0.008 | 0.055 | 0.182 | 0.089 | 0.064 | 0.080 |
| 124 | 0.174 | 0.158 | 0.323 | 0.276 | 0.149 | 0.018 | 0.013 | 0.007 | 0.001 | 0.008 | 0.052 | 0.176 | 0.097 | 0.060 | 0.076 |
| 125 | 0.179 | 0.166 | 0.348 | 0.280 | 0.179 | 0.015 | 0.011 | 0.007 | 0.001 | 0.006 | 0.055 | 0.163 | 0.085 | 0.064 | 0.077 |
| 126 | 0.154 | 0.174 | 0.351 | 0.243 | 0.179 | 0.017 | 0.011 | 0.008 | 0.002 | 0.007 | 0.055 | 0.157 | 0.098 | 0.066 | 0.072 |
| 127 | 0.174 | 0.150 | 0.395 | 0.290 | 0.177 | 0.015 | 0.012 | 0.007 | 0.001 | 0.009 | 0.054 | 0.173 | 0.089 | 0.062 | 0.076 |
| 128 | 0.182 | 0.148 | 0.346 | 0.281 | 0.134 | 0.018 | 0.014 | 0.006 | 0.001 | 0.008 | 0.051 | 0.164 | 0.085 | 0.051 | 0.073 |
| 129 | 0.162 | 0.164 | 0.393 | 0.282 | 0.163 | 0.015 | 0.009 | 0.006 | 0.001 | 0.008 | 0.051 | 0.143 | 0.086 | 0.056 | 0.076 |
| 130 | 0.147 | 0.153 | 0.330 | 0.265 | 0.144 | 0.017 | 0.008 | 0.007 | 0.001 | 0.008 | 0.054 | 0.186 | 0.086 | 0.056 | 0.074 |
| 131 | 0.175 | 0.155 | 0.280 | 0.270 | 0.163 | 0.014 | 0.012 | 0.008 | 0.001 | 0.008 | 0.049 | 0.187 | 0.086 | 0.061 | 0.078 |
| 132 | 0.152 | 0.133 | 0.333 | 0.274 | 0.167 | 0.019 | 0.008 | 0.009 | 0.001 | 0.008 | 0.052 | 0.160 | 0.074 | 0.054 | 0.077 |
| 133 | 0.152 | 0.173 | 0.325 | 0.255 | 0.167 | 0.016 | 0.009 | 0.006 | 0.001 | 0.008 | 0.044 | 0.154 | 0.091 | 0.054 | 0.056 |
| 134 | 0.129 | 0.148 | 0.362 | 0.229 | 0.159 | 0.013 | 0.009 | 0.007 | 0.001 | 0.007 | 0.056 | 0.176 | 0.078 | 0.058 | 0.069 |
| 135 | 0.151 | 0.120 | 0.321 | 0.230 | 0.169 | 0.014 | 0.010 | 0.003 | 0.001 | 0.009 | 0.049 | 0.165 | 0.076 | 0.060 | 0.065 |
| 136 | 0.157 | 0.141 | 0.332 | 0.228 | 0.159 | 0.013 | 0.009 | 0.007 | 0.001 | 0.009 | 0.046 | 0.164 | 0.078 | 0.054 | 0.056 |
| 137 | 0.150 | 0.136 | 0.282 | 0.256 | 0.133 | 0.013 | 0.008 | 0.006 | 0.001 | 0.008 | 0.047 | 0.177 | 0.081 | 0.052 | 0.066 |
| 138 | 0.167 | 0.142 | 0.310 | 0.197 | 0.133 | 0.012 | 0.010 | 0.008 | 0.001 | 0.008 | 0.043 | 0.170 | 0.071 | 0.054 | 0.073 |
| 139 | 0.161 | 0.137 | 0.337 | 0.238 | 0.147 | 0.017 | 0.011 | 0.008 | 0.001 | 0.005 | 0.043 | 0.170 | 0.077 | 0.047 | 0.063 |
| 140 | 0.141 | 0.115 | 0.298 | 0.249 | 0.147 | 0.014 | 0.011 | 0.006 | 0.001 | 0.007 | 0.048 | 0.184 | 0.074 | 0.045 | 0.064 |
| 141 | 0.140 | 0.126 | 0.291 | 0.210 | 0.136 | 0.014 | 0.013 | 0.006 | 0.001 | 0.007 | 0.046 | 0.152 | 0.070 | 0.040 | 0.064 |
| 142 | 0.146 | 0.126 | 0.307 | 0.207 | 0.143 | 0.013 | 0.010 | 0.007 | 0.001 | 0.008 | 0.050 | 0.171 | 0.065 | 0.047 | 0.058 |
| 143 | 0.139 | 0.132 | 0.301 | 0.221 | 0.155 | 0.015 | 0.010 | 0.005 | 0.001 | 0.007 | 0.047 | 0.126 | 0.066 | 0.047 | 0.058 |
| 144 | 0.127 | 0.116 | 0.290 | 0.217 | 0.140 | 0.013 | 0.011 | 0.006 | 0.001 | 0.009 | 0.037 | 0.166 | 0.065 | 0.043 | 0.053 |
| 145 | 0.140 | 0.115 | 0.331 | 0.209 | 0.138 | 0.012 | 0.011 | 0.004 | 0.001 | 0.008 | 0.047 | 0.146 | 0.069 | 0.040 | 0.053 |
| 146 | 0.145 | 0.127 | 0.243 | 0.186 | 0.137 | 0.015 | 0.010 | 0.005 | 0.001 | 0.007 | 0.043 | 0.160 | 0.067 | 0.039 | 0.052 |
| 147 | 0.127 | 0.111 | 0.279 | 0.222 | 0.111 | 0.014 | 0.010 | 0.007 | 0.001 | 0.007 | 0.041 | 0.154 | 0.072 | 0.035 | 0.058 |
| 148 | 0.129 | 0.107 | 0.236 | 0.202 | 0.126 | 0.015 | 0.010 | 0.005 | 0.001 | 0.007 | 0.042 | 0.160 | 0.071 | 0.036 | 0.055 |
| 149 | 0.123 | 0.117 | 0.287 | 0.220 | 0.105 | 0.012 | 0.012 | 0.007 | 0.001 | 0.008 | 0.039 | 0.167 | 0.070 | 0.037 | 0.058 |
| 150 | 0.125 | 0.127 | 0.270 | 0.200 | 0.128 | 0.013 | 0.008 | 0.007 | 0.001 | 0.007 | 0.042 | 0.139 | 0.067 | 0.039 | 0.059 |

**Table S2**. The kinetic data of five competition patients used in Phases 1 and 2 of the challenge.

|  | **% Administered Activity** | | | | | **% Administered Activity/Volume** | | | | | **% Administered Activity** | | | | |
| --- | --- | --- | --- | --- | --- | --- | --- | --- | --- | --- | --- | --- | --- | --- | --- |
| **Hours p.i.** | **Kidneys** | | | | | **Tumor** | | | | | **Blood** | | | | |
|  | **P6** | **P7** | **P8** | **P9** | **P10** | **P6** | **P7** | **P8** | **P9** | **P10** | **P6** | **P7** | **P8** | **P9** | **P10** |
| 1.4 | 1.604 | 1.794 | 1.948 | 3.827 | 2.067 | 0.039 | 0.018 | 0.082 | 0.003 | 0.027 | 13.423 | 10.489 | 13.847 | 9.072 | 8.036 |
| 18.5 | 1.228 | 1.295 | 0.901 | 2.673 | 1.319 | 0.052 | 0.031 | 0.073 | 0.005 | 0.019 | 3.003 | 1.882 | 1.160 | 1.443 | 0.362 |
| 42.2 | 0.700 | 0.745 | 0.627 | 1.588 | 0.714 | 0.047 | 0.032 | 0.053 | 0.004 | 0.011 | 0.989 | 0.472 | 0.281 | 0.294 | 0.091 |
| 65.8 | 0.402 | 0.533 | 0.413 | 0.959 | 0.405 | 0.029 | 0.024 | 0.048 | 0.002 | 0.005 | 0.397 | 0.192 | 0.095 | 0.092 | 0.046 |
| 138.4 | 0.115 | 0.160 | 0.148 | 0.243 | 0.120 | 0.011 | 0.014 | 0.030 | 0.002 | 0.003 | 0.062 | 0.044 | 0.034 | 0.017 | 0.020 |

**Table S3**. Ground truth TIAC values for the five patients used in phase 0 of the challenge.

| **Kidneys TIAC [h]** | | | | | **Tumor TIAC [h/cc]** | | | | | **Blood TIAC [h]** | | | | |
| --- | --- | --- | --- | --- | --- | --- | --- | --- | --- | --- | --- | --- | --- | --- |
| **P1** | **P2** | **P3** | **P4** | **P5** | **P1** | **P2** | **P3** | **P4** | **P5** | **P1** | **P2** | **P3** | **P4** | **P5** |
| 0.786 | 0.628 | 0.176 | 0.122 | 0.612 | 0.584 | 0.398 | 0.239 | 0.705 | 0.260 | 0.963 | 0.875 | 0.134 | 0.243 | 0.178 |

**Table S4.** Ground truth TIAC values for the five patients used in phase 1 and phase 2 of the challenge.

| **Kidneys TIAC [h]** | | | | | **Tumor TIAC [h/cc]** | | | | | **Blood TIAC [h]** | | | | |
| --- | --- | --- | --- | --- | --- | --- | --- | --- | --- | --- | --- | --- | --- | --- |
| **P1** | **P2** | **P3** | **P4** | **P5** | **P1** | **P2** | **P3** | **P4** | **P5** | **P1** | **P2** | **P3** | **P4** | **P5** |
| 0.846 | 0.925 | 0.806 | 1.934 | 0.958 | 0.062 | 0.049 | 0.124 | 0.005 | 0.019 | 1.951 | 1.195 | 1.088 | 0.925 | 0.513 |

**Functions used to create the synthetic data**


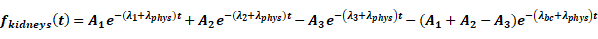


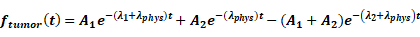


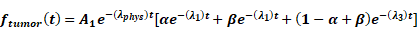


where fi is a fit function with i being the organ of interest,
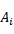

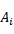
 are the coefficients of the respective exponential terms,
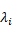

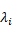
 are the biological clearance or uptake rates of the radiopharmaceutical,
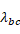

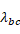
 is the rate of blood circulation set to the half-life of 1 min, alpha and beta are the fractions of the exponential terms, and
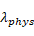

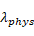
 is the physical decay constant of ^177^Lu (6.6443 d). For the noise simulations, a 10 % standard deviation proportional error was used.

Appendix 2: Questionnaire for phases 0 to 2 of the challenge

| **Question** | |
| --- | --- |
| G1. | Team name |
| G2. | TACTIC phase (e.g., 0,1 or 2) |
| G3. | Contact email |
| **Kidney** | |
| K.1 | Please insert the formula of the function you used, including constant values |
| K.2 | Provide all fit results, e.g. parameter values, for all patients within this organ. |
| K.3.a | Have you incorporated a data uncertainty model into the fit? |
| K.3.b | If yes: Which one? Please define and give arguments/reasons |
| K.4 | Which Objective Function did you use (e.g., least squares)? |
| K.5.a | How did you check the goodness of fits? |
| K.5.b | Here, we would like you to unambiguously define your method to check the goodness of fits |
| K.6.a | Was population information taken into account in the used algorithm? |
| K.6.b | If yes: How was the population information incorporated into the fit model? |
| K.7 | Any additional information needed to reproduce your results can be added here |
| **Blood** | |
| B.1 | Please insert the formula of the function you used, including constant values |
| B.2 | Provide all fit results, e.g. parameter values, for all patients within this organ. |
| B.3.a | Have you incorporated a data uncertainty model into the fit? |
| B.3.b | If yes: Which one? Please define and give arguments/reasons |
| B.4 | Which Objective Function did you use (e.g., least squares)? |
| B.5.a | How did you check the goodness of fits? |
| B.5.b | Here, we would like you to unambiguously define your method to check the goodness of fits |
| B.6.a | Was population information taken into account in the used algorithm? |
| B.6.b | If yes: How was the population information incorporated into the fit model? |
| B.7 | Any additional information needed to reproduce your results can be added here |
| **Tumor** | |
| T.1 | Please insert the formula of the function you used, including constant values |
| T.2 | Provide all fit results, e.g. parameter values, for all patients within this organ. |
| T.3.a | Have you incorporated a data uncertainty model into the fit? |
| T.3.b | If yes: Which one? Please define and give arguments/reasons |
| T.4 | Which Objective Function did you use (e.g., least squares)? |
| T.5.a | How did you check the goodness of fits? |
| T.5.b | Here, we would like you to unambiguously define your method to check the goodness of fits |
| T.6.a | Was the population information taken into account in the used algorithm? |
| T.6.b | If yes: How was the population information incorporated into the fit model? |
| T.7 | Any additional information needed to reproduce your results can be added here |

Appendix 3: Leaderboard of the 1^st^ competition phase

**Table S4**. Leaderboard of the 1^st^ competition phase: fitting using individual biokinetic data

| **#** | **Team/participant (university*)** | **RMSE sum** | **Organ TIAC RMSE** | | |
| --- | --- | --- | --- | --- | --- |
|  |  |  | **Kidney [h]** | **Tumor [h/cc]** | **Blood [h]** |
| **1** | **Centro di Riferimento Oncologico di Aviano (CRO) IRCCS** | **0.162** | **0.042** | **0.010** | **0.110** |
| 2 | University of Western Ontario | 0.184 | 0.065 | 0.007 | 0.112 |
| 3 | MD Anderson | 0.192 | 0.076 | 0.009 | 0.107 |
| 4 | Cleveland Clinic | 0.193 | 0.046 | 0.017 | 0.130 |
| 5 | University Medical Center Hamburg-Eppendorf | 0.194 | 0.080 | 0.007 | 0.107 |
| 6 | Charité – Universitätsmedizin Berlin | 0.197 | 0.097 | 0.011 | 0.089 |
| 7 | Dosicro (Invicro) | 0.198 | 0.093 | 0.013 | 0.092 |
| 8 | University of South Carolina | 0.200 | 0.038 | 0.008 | 0.154 |
| 9 | RTX (Ratio Therapeutics) | 0.202 | 0.089 | 0.011 | 0.102 |
| 10 | UKA (University Hospital Augsburg) | 0.204 | 0.047 | 0.010 | 0.147 |
| 11 | Washington University in St. Louis | 0.219 | 0.054 | 0.011 | 0.154 |
| 12 | Fondazione Poliambulanza | 0.223 | 0.063 | 0.011 | 0.148 |
| 13 | Memorial Sloan Kettering Cancer Center | 0.223 | 0.067 | 0.009 | 0.147 |
| 14 | University of Leipzig Medical Centre | 0.223 | 0.055 | 0.015 | 0.153 |
| 15 | Azienda Ospedaliero-Universitaria Pisana | 0.230 | 0.100 | 0.006 | 0.125 |
| 16 | Loyola University Medical Center | 0.251 | 0.089 | 0.028 | 0.134 |
| 17 | LEDI (L'Institut de Radioprotection et de Sûreté Nucléaire) | 0.263 | 0.108 | 0.009 | 0.147 |
| 18 | Université de Montréal | 0.302 | 0.188 | 0.011 | 0.102 |
| 19 | European Radiation Dosimetry Group | 0.320 | 0.046 | 0.009 | 0.265 |
| 20 | Helsinki University Hospital | 0.336 | 0.055 | 0.013 | 0.267 |
| 21 | Oregon Health & Science University | 0.357 | 0.074 | 0.010 | 0.272 |
| 22 | University of Alabama at Birmingham | 0.362 | 0.256 | 0.009 | 0.097 |
| 23 | UC Davis | 0.399 | 0.085 | 0.028 | 0.286 |
| 24 | TORVE (Homepage Università degli Studi di Roma Tor Vergata) | 0.402 | 0.127 | 0.027 | 0.248 |
| 25 | Texas Children’s Hospital | 0.452 | 0.151 | 0.165 | 0.136 |
| 26 | University of Alberta | 0.480 | 0.052 | 0.006 | 0.421 |
| 27 | Princess Margaret Cancer Centre | 0.513 | 0.120 | 0.006 | 0.386 |
| 28 | Tirol Kliniken | 0.559 | 0.065 | 0.009 | 0.484 |
| 29 | University of Chicago | 0.660 | 0.051 | 0.019 | 0.590 |
| 30 | Pontifícia Universidade Católica do Rio Grande do Sul | 0.670 | 0.172 | 0.014 | 0.485 |
| 31 | Karolinska University | 1.199 | 0.097 | 0.008 | 1.095 |
| 32 | Rhode Island Hospital, Brown University | 1.533 | 1.066 | 0.324 | 0.143 |
| 33 | University of Minnesota | 1.687 | 0.100 | 0.021 | 1.566 |

*optional information to share

Appendix 4: Objective functions

**Table S5**. Objective functions used in various phases of the TACTIC challenge.

| **Objective function type** | **Frequency of use within the phase** | | |
| --- | --- | --- | --- |
|  | **Phase 0** | **Phase 1** | **Phase 2** |
| Least Square Error | 19 | 15 | 12 |
| Non-linear least Square Error | 2 | 4 | 1 |
| Non-linear Mean Error | 0 | 0 | 6 |
| R2 | 2 | 1 | 0 |
| Maximum likelihood expectation | 2 | 0 | 0 |
| Standardized Square Error | 2 | 2 | 0 |
| Chi-square error | 2 | 2 | 0 |
| Mean Square Error | 2 | 1 | 1 |
| Logg-error | 1 | 0 | 0 |
| N/A | 2 | 2 | 5 |
| L2-error | 0 | 1 | 1 |
| Reduced Chi-square | 0 | 1 | 1 |
| Trust Region Reflective algorithm | 0 | 1 | 0 |
| SAEM | 0 | 1 | 1 |
| Root Mean Square Error | 0 | 1 | 0 |

Appendix 5: Leaderboard of the 2^nd^ competition phase

**Table S6**. Leaderboard of the 2^nd^ competition phase: fit model incorporating population-based biokinetic data

| **#** | **Team/participant (university*)** | **RMSE sum** | **Organ TIAC RMSE** | | |
| --- | --- | --- | --- | --- | --- |
|  |  |  | **Kidney [h]** | **Tumor [h/cc]** | **Blood [h]** |
| 1 | **Cleveland Clinic** | **0.117** | **0.040** | **0.020** | **0.058** |
| 2 | University of Leipzig Medical Centre | 0.148 | 0.066 | 0.015 | 0.067 |
| 3 | European Radiation Dosimetry Group | 0.160 | 0.028 | 0.011 | 0.121 |
| 4 | Centro di Riferimento Oncologico di Aviano (CRO) IRCCS | 0.165 | 0.042 | 0.008 | 0.115 |
| 5 | University of South Carolina | 0.176 | 0.038 | 0.008 | 0.131 |
| 6 | Helsinki University Hospital | 0.177 | 0.074 | 0.007 | 0.096 |
| 7 | MD Anderson | 0.192 | 0.076 | 0.009 | 0.107 |
| 8 | University Medical Center Hamburg-Eppendorf | 0.194 | 0.080 | 0.007 | 0.107 |
| 9 | University Hospital Augsburg | 0.204 | 0.047 | 0.010 | 0.147 |
| 10 | Alepi | 0.206 | 0.047 | 0.006 | 0.152 |
| 11 | RTX (Ratio Therapeutics) | 0.213 | 0.086 | 0.012 | 0.116 |
| 12 | Memorial Sloan Kettering Cancer Center | 0.223 | 0.067 | 0.009 | 0.147 |
| 13 | University of Western Ontario | 0.228 | 0.075 | 0.006 | 0.146 |
| 14 | Fondazione Poliambulanza | 0.229 | 0.075 | 0.006 | 0.148 |
| 15 | Princess Margaret Cancer Centre | 0.231 | 0.066 | 0.013 | 0.153 |
| 16 | MGH, Harvard University | 0.241 | 0.051 | 0.005 | 0.185 |
| 17 | Washington University in St. Louis | 0.246 | 0.055 | 0.011 | 0.180 |
| 18 | University of Alabama at Birmingham | 0.475 | 0.475 |  |  |
| 19 | University of Alberta | 0.480 | 0.052 | 0.006 | 0.421 |
| 20 | LEDI ’L'Institut de Radioprotection et de Sûreté Nucléaire) | 0.534 | 0.378 | 0.011 | 0.145 |
| 21 | University of Chicago | 0.860 | 0.066 | 0.016 | 0.778 |
| 22 | University of Minnesota | 0.870 | 0.240 | 0.020 | 0.609 |
| 23 | UC Davis | 0.927 | 0.547 | 0.061 | 0.319 |
| 24 | Oregon Health & Science University | 0.957 | 0.057 | 0.017 | 0.883 |
| 25 | Loyola University Medical Center | 0.999 | 0.549 | 0.101 | 0.349 |
| 26 | Rhode Island Hospital, Brown University | 1.104 | 0.517 | 0.070 | 0.517 |
| 27 | Sajid Bashir | 2.208 | 0.626 | 0.446 | 1.136 |
| 28 | Pontifícia Universidade Católica do Rio Grande do Sul | 4.069 | 0.304 | 3.107 | 0.658 |

*optional information to share
